# Bioactive Compounds of Sea Mustard (*Undaria pinnatifida*) Waste Affected by Drying Methods

**DOI:** 10.3390/foods13233815

**Published:** 2024-11-26

**Authors:** Rea Mae Templonuevo, Kang-Hee Lee, Seung-Min Oh, Yue Zhao, Jiyeon Chun

**Affiliations:** 1 Department of Food Science and Technology, Sunchon National University, Suncheon 57922, Jeonnam, Republic of Korea; reamaetemplonuevo@clsu.edu.ph (R.M.T.); nw1526@naver.com (K.-H.L.); dta1532@naver.com (S.-M.O.); czy20220405@gmail.com (Y.Z.); 2 College of Fisheries, Central Luzon State University, Science City of Muñoz 3120, Nueva Ecija, Philippines; 3 Bio-Healthcare Research and Analysis Center, Sunchon National University, Suncheon 57922, Jeonnam, Republic of Korea; 4 Glocal University Project Team, Sunchon National University, 255 Jungangno, Suncheon 57922, Jeonnam, Republic of Korea

**Keywords:** sea mustard waste parts, bioactive compounds, antioxidant properties

## Abstract

Sea mustard (*Undaria pinnatifida*) is a brown macroalga extensively cultivated and consumed in South Korea. However, the high volume of seaweed production in the country results in substantial waste generation. To mitigate this issue, the bioactive compounds of sea mustard waste parts (sporophyll, root, and stem) were assessed under different drying conditions (freeze, oven, and microwave drying) to evaluate their potential as functional ingredients. The sporophyll contained the highest levels of total chlorophyll (540.38 μg/g), fucoxanthin (165.87 μg/g), flavonoids (5.47 μg QE/g), phytomenadione (332.59 μg/100 g), and cobalamin (5.92 μg/100 g). In contrast, the root exhibited the highest antioxidant activities (DPPH: 1582.37 μg GAE/g; ABTS: 0.93 mg AAE/g), total polyphenol (2718.81 μg GAE/g) and phlorotannin (4298.22 μg PGE/g) contents. Freeze drying achieved the best retention rates for most bioactive compounds, except for fucoxanthin, which was highest in microwave-dried samples. These results demonstrate the potential of sea mustard waste as a valuable source of bioactive compounds, with the retention of these compounds being influenced by drying methods, depending on the specific part of the seaweed.

## 1. Introduction

Seaweeds are among the top aquaculture products worldwide and are known for their rich composition of structural molecules such as proteins, lipids, and carbohydrates. They are also a source of various bioactive compounds, including phycocolloids, polysaccharides, unsaturated fatty acids, fiber, vitamins, and minerals. These compounds are utilized in the production of diverse products in the food, cosmetics, agriculture, and nutraceutical industries [1]. Numerous studies have reported the health and nutritional benefits of seaweeds, making them a valuable ingredient in novel food and health-related products [2]. As a result, global demand for seaweed has steadily increased over the years.

The large-scale production of seaweed generates significant amounts of waste after harvest. This waste is primarily produced during the extraction of industrial compounds such as alginates and carrageenan, which are widely used as thickening and emulsifying agents [3]. For instance, the extraction yield of alginates ranges from 17% to 49.9%, leaving behind approximately 50% as waste [4]. Additionally, parts of seaweed not used for compound extraction or food processing are often discarded. While some of these solid wastes are transformed into low-market-value products such as plant fertilizers or animal feed [5], a substantial proportion is disposed of in landfills or the ocean [6]. This practice contributes to marine pollution, increases carbon emissions, and negatively impacts seaweed farming in subsequent years, thereby reducing production.

South Korea, one of the world’s leading seaweed producers, faces challenges related to high energy consumption and carbon emissions [7]. According to the Ministry of Oceans and Fisheries (2023) [8], seaweed waste has the lowest recycling rate (0%) among the country’s top fishery products. Given the scale of seaweed production, the efficient management of seaweed waste is critical for protecting the marine environment and recycling bioactive substances. This waste can potentially be transformed into functional ingredients for applications aimed at enhancing the health-promoting properties of various products.

*Undaria pinnatifida*, commonly known as sea mustard, is a brown seaweed endemic to Korea and widely cultivated in the region. In 2017, South Korea produced over 622,613 tons of *U. pinnatifida*, accounting for 35% of the country’s total seaweed production [9]. Due to its excellent taste and health benefits, including antioxidant, anticancer, antihypertensive, antidiabetic, and antihypercholesterolemic properties, it has gained substantial consumer interest [10]. Sea mustard is commonly served as a side dish in Korean cuisine and is widely available in dried form. However, since the leaf is the most consumed part, large quantities of waste are generated, comprising sporophylls, roots, and stems. For example, roots account for 40–60% of annual waste production at farming sites [11].

Considering that seaweed naturally contains diverse bioactive compounds, these underutilized waste parts may also have significant value. Some studies have focused on extracting bioactive compounds, such as fucoidan and alginates, from sporophylls. However, limited research has examined the bioactive potential of roots and stems, which are typically discarded as waste. Moreover, environmental factors can influence the nutrient composition of seaweeds, adding variability to their bioactive properties.

Fresh seaweed, characterized by its high moisture content, is commonly dried as a pre-treatment step before industrial processing. Drying is a critical process that reduces water activity, thereby inhibiting microbial growth and extending shelf life [12]. Traditional methods, such as sun drying and oven drying, have been utilized for centuries. Freeze drying, although effective in preserving quality through low-temperature processing, is associated with prolonged processing times and high capital costs. Alternatively, microwave drying offers a faster and more efficient process but involves higher temperatures, which may cause adverse chemical changes in the product.

Drying processes often induce enzymatic and non-enzymatic reactions, leading to some alterations in the nutritional and functional properties of seaweeds. Furthermore, different parts of seaweed exhibit varied responses to drying methods due to their distinct structural compositions. Determining drying conditions for specific industrial applications is, therefore, essential to maximize the potential of seaweeds. This study investigates the effects of different drying methods on the health-promoting components and antioxidant properties of *U. pinnatifida* waste parts, including roots, sporophylls, and stems. The findings aim to evaluate the feasibility of these waste parts as functional ingredients for applications in the food and nutraceutical industries.

## 2. Materials and Methods

### 2.1. Materials

Standard chemicals including gallic acid, ascorbic acid, quercetin, phloroglucinol, phylloquinone, menaquinone, and cyanocobalamin were obtained from Sigma-Aldrich (St. Louis, MO, USA). All other reagents and solvents utilized in the study were of ACS or high-performance liquid chromatography (HPLC) grades. The commercial infant formula (Imperial Dream XO World Class 3, Namyang, Seoul, Korea) used as an analytical quality control sample was purchased from a local supermarket and stored at −70 °C.

### 2.2. Sample Preparation

Sea mustard waste parts were obtained from Bada & Haecho Fishery Corp. (Goheung, Jeonnam, Korea). These were harvested between March and May 2023 from the coastal waters of Goheung, Jeollanam-do province, South Korea. The samples were thoroughly washed under running water to remove debris, then drained, sealed in polyethylene bags, and stored at −70 °C until the drying processes were carried out.

**Figure 1:**
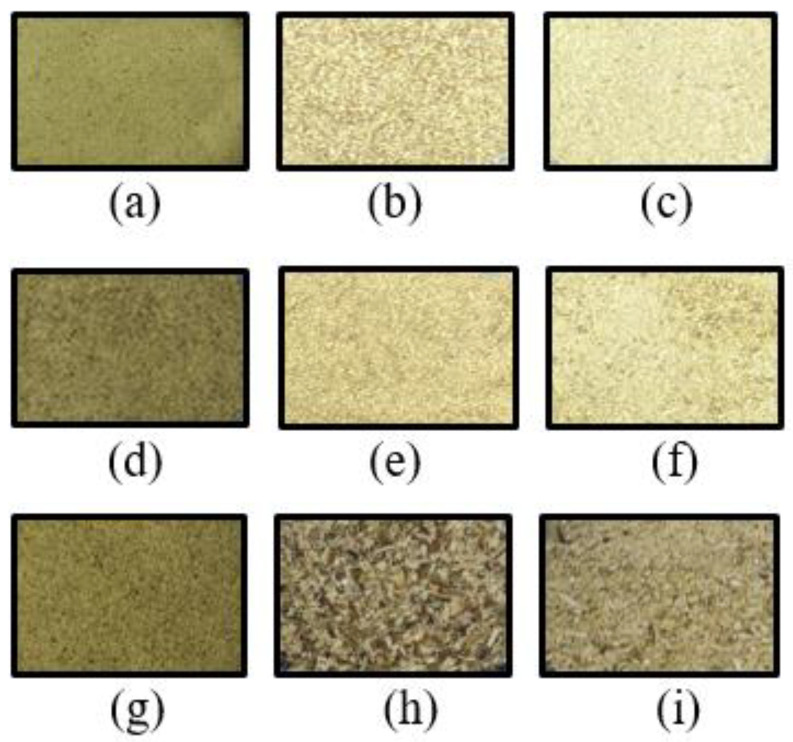
Images of dried sea mustard waste parts: (**a**) freeze-dried sporophyll; (**b**) freeze-dried root; (**c**) freeze-dried stem; (**d**) oven-dried sporophyll; (**e**) oven-dried root; (**f**) oven-dried stem; (**g**) microwave-dried sporophyll; (**h**) microwave-dried root; (**i**) microwave-dried stem.

The sea mustard waste parts were dried under three different conditions: freeze drying at −70 °C (Il-Shin Freeze Dryer Series, Il-Shinbiobase Co., Ltd., Yangju, Korea), oven drying (HB-502M, HanBaek Scientific Co., Bucheon, Korea) at 40 °C, and microwave drying at 260 W (ER-4320B, LG Electronics Tianjin Appliance s Co., Ltd., Seoul, Korea). Drying was carried out until the equilibrium moisture content was reached for each part of the sea mustard: <18% for sporophyll and <12% for both stem and root. Equilibrium moisture content was defined as the point at which the samples maintained a constant moisture level over time. The dried seaweed was ground using a blender (HMF-3250S; Hanil Science Industrial Co., Gwangju, Korea), sieved (Laboratory Test Sieve No. 50) at 300 μm (Figure 1), and stored at −70 °C until assay.

### 2.3. Total Fucoxanthin Analysis

Total fucoxanthin content was measured using the method of Osório et al. [13] with some modifications. Briefly, 100 mg of dry powdered sample was added with 10 mL of DMSO-water (4:1, *v*/*v*) and vortexed for 1 min. The extract was then centrifuged at 700× *g* (MF-550, Hanil Science Co., Gwangju, Korea) for 10 min and the supernatant was subjected to another centrifugation at 1413.2× *g* for 5 min. Absorbance was measured using a microplate reader (Eon, BioTek Instruments, Winooski, VT, USA) at 480, 582, 631, 665, and 750 nm against a DMSO-water blank. Total fucoxanthin content was calculated using the formula below.

Fucoxanthin (μg/mL) = 7.69 × (A480 − A750) − 5.55 × [(A631 − A750) + (A582 − A750) − 0.297 × (A665 − A750)] − 0.377 × (A665 − A750)(1)

### 2.4. Chlorophyll Analyses

Chlorophylls were measured using the method of Osório et al. [13] with some modifications. Briefly, 100 mg of dry powdered sample was mixed with 10 mL of 90% acetone and vortexed for 1 min. The extract was then centrifuged at 700× *g* (MF-550, Hanil Science Co.) for 10 min, and the collected supernatant was subjected to another centrifugation at 1413.2× *g* for 5 min. Absorbance was measured at 630, 647, 664, 691, and 750 nm against the solvent (90% acetone) blank using a microplate reader. Values were computed using the following formula.

Chl a (μg/mL) = −0.3319 × (A630 − A750) − 1.7485 × (A647 − A750) + 11.9442 × (A664 − A750) − 1.4306 × (A691 − A750) (±0.0020)(2)

Chl b (μg/mL) = −1.2825 × (A630 − A750) − 19.8839 × (A647 − A750) − 4.8860 × (A664 − A750) − 2.3416 × (A691 − A750) (±0.0076)(3)

Chl c (μg/mL) = 23.5902 × (A630 − A750) − 7.8516 × (A647 − A750) − 1.5214 × (A664 − A750) − 1.7443 × (A691 − A750) (±0.0075)(4)

Chl d (μg/mL) = −0.5881 × (A630 − A750) + 0.0902 × (A647 − A750) − 0.1564 × (A664 − A750) + 11.0473 × (A691 − A750) (±0.0030)(5)

Total Chl (μg/mL) = Chl a + Chl b + Chl c + Chl d(6)

### 2.5. DPPH Radical Scavenging Activity

The DPPH radical scavenging activity was measured using the method of Blois [14], with some modifications. Sixty microliters of the sample extract was mixed with 240 μL of 0.02 mM DPPH solution and allowed to react in the dark for 30 min. The DPPH solution was prepared by dissolving 0.00789 g of DPPH reagent in 100 mL of 70% ethanol. Absorbance was measured at 517 nm using a microplate reader. The standard used was gallic acid and the unit of values were expressed as gallic acid equivalents in μg/g. The concentrations used for the calibration curve were 0, 2, 4, 8, 12, 16, and 20 μg/mL.

### 2.6. ABTS Radical Scavenging Activity

ABTS radical scavenging activity was analyzed using the method of Arts et al. [15] with some modifications. Fifteen microliters of the sample extract was mixed with 285 μL of ABTS solution, prepared by dissolving ABTS reagent in 2.45 mM potassium persulfate buffer, and allowed to react in the dark for 7 min. Absorbance was measured at 734 nm using a microplate reader. Ascorbic acid was used as the standard, and results were expressed as ascorbic acid equivalents in mg/g. The concentrations used for the calibration curve were 0, 20, 40, 60, 80, and 100 μg/mL.

### 2.7. Total Polyphenol Analysis

The total polyphenol content was measured using the method of Singleton et al. [16] with some modifications. Forty microliters of the sample extract was mixed with 200 μL of distilled water and 20 μL of Folin reagent. After 3 min, 40 μL of Na₂NO₃ was added, and the mixture was allowed to react in the dark for 1 h. Absorbance was measured at 750 nm using a microplate reader. Gallic acid was used as the standard, and results were expressed as gallic acid equivalents in μg/g. The concentrations used for the calibration curve were 0, 20, 40, 60, 80, and 100 μg/mL.

### 2.8. Total Flavonoid Analysis

Total flavonoid content was analyzed using the method of Zhishen et al. [17] with some modifications. One hundred microliters of the sample extract was mixed with 400 µL of 80% ethanol and 30 µL of 5% NaNO₂. The mixture was vortexed for 30 s and allowed to react for 5 min. Next, 30 µL of 10% AlCl₃ was added, and the reaction was allowed to proceed for another 5 min. Finally, 200 µL of double-distilled water was added, and the solution was vortexed for 30 s. Absorbance was measured at 420 nm using a microplate reader. Quercetin was used as the standard, and results were expressed as quercetin equivalents in μg/g. The concentrations used for the calibration curve were 0, 100, 200, 400, 600, 800, and 1000 μg/mL.

### 2.9. Total Phlorotannin Analysis

The total phlorotannin content was measured using the method of Li et al. [18] with some modifications. Twenty microliters of the sample extract was mixed with 130 µL of double-distilled water. Then, 50 µL of Folin solution and 100 µL of sodium carbonate solution were added and allowed to react in the dark for 1 h. Absorbance was measured at 770 nm using a microplate reader. Phloroglucinol was used as the standard, and results were expressed as phloroglucinol equivalents in μg/g. The concentrations used for the calibration curve were 0, 20, 40, 60, 80, and 100 μg/mL.

### 2.10. Phytomenadione Analysis

Phytomenadione (phylloquinone, vitamin K_1_) analysis was carried out using the method outlined in the Food Code by the Ministry of Food and Drug Safety [19] with some modifications. One gram of sea mustard sample was mixed with 30 mL of dichloromethane and methanol (2:1) solution. Extraction was performed using an ultrasonic extractor (117 volts, 47 kHz) for 1 hr. The solution was then filtered through Whatman No.2 filter paper (8 μm) (Cytiva, Marlborough, MA, USA) with anhydrous sodium sulfate. The filtrate was collected in a volumetric flask and diluted with methanol to a final volume of 50 mL. This extract was used for HPLC analysis.

Separation and qualification of phytomenadione were performed by using HPLC-fluorescence detection [20]. Briefly, 2 mL of sample extract was placed in a test tube and evaporated under nitrogen gas at 45 °C. Then, exactly 2 mL of hexane was added and vortexed to re-dissolve the extract. Next, 5 mL of clean-up solvent (methanol–water = 9:1, *v*/*v*) was added and vortexed. The mixture was centrifuged at 353.3× *g* for 5 min. The upper layer was transferred to a separate test tube and dried under nitrogen gas at 45 °C. Exactly 1 mL of methanol was added, and the solution was vortexed. The solution was then filtered using a syringe filter (13 mm, 0.45 μm, FUTEC Co., Ltd., Daejon, Korea) and injected into the HPLC system equipped with a fluorescence detector (Agilent, 1200 series, Santa Clara, CA, USA). Phytomenadione was separated using a ZORBAX Eclipse XDB-C18 column (5 μm, 4.6 × 150 mm, Agilent), which was connected to a post-column (2.0 mm × 50 mm, YMC Co., Ltd., Kyoto, Japan). The excitation and emission wavelengths used were 243 nm and 430 nm, respectively. The following conditions were applied during the analysis: flow rate, 1.0 mL/min; column oven temperature, 35 °C; and injection volume, 20 μL. The isocratic mobile phase consisted of a mixed solution of methanol–dichloromethane (9:1, *v*/*v*) containing 3 mM sodium acetate trihydrate, 10 mM zinc chloride, and 5 mM acetic acid.

### 2.11. Cobalamin Analyses

Cobalamins (vitamin B_12_) in the seaweed wastes were analyzed using the method of Park et al. [21]. Two grams of sample were mixed with 0.5 mL of 1% sodium cyanide and 49.5 mL of 0.2 M sodium acetate trihydrate buffer (pH 4.0), then sonicated for 10 min using an ultrasonic homogenizer (8893-DHT, Cole-Parmer, Chicago, IL, USA). The mixture was then heated for 1 hr in a water bath (WB-20M, Jeio Tech Co., Daejeon, Korea) to extract cobalamins and convert into cyanocobalamin. The extracted samples were cooled and filtered through Whatman No. 1 filter paper (185 mm, Cytiva). The filtered samples were then used for analysis.

The extracted samples were purified and concentrated using an immunoaffinity column (Easi-Extract Vitamin B_12_, R-Biopharm Rhone Ltd., Glasgow, UK). The refrigerated immunoaffinity column was first stabilized at room temperature, and the buffer solution inside was removed, followed by conditioning with 3 mL of water. Next, 9 mL of the extracted sample was injected to adsorb cyanocobalamin onto the immune-reactive gel inside the column. The column was washed with 9 mL of water to remove impurities. The remaining moisture inside the column was removed using a syringe, and 3 mL of methanol was injected to elute cyanocobalamin. The eluted extract was evaporated completely under nitrogen at 70 °C, reconstituted with 0.5 mL of water, filtered through a 0.45 μm membrane filter (Futecs Co., Daejeon, Korea), and used for HPLC analysis.

The separation and quantification of cyanocobalamin were performed using an HPLC system (1260 infinity, Agilent, Santa Clara, CA, USA) equipped with a C18 ACE 3 AQ column (3 mm × 150 mm, ACE, Aberdeen, Scotland) and a PDA detector. The mobile phase consisted of water and acetonitrile, with the gradient conditions shown in Table 1. Other analytical conditions were as follows: UV detection at 361 nm, flow rate of 0.25 mL/min, column oven temperature of 35 °C, and an injection volume of 100 μL.

### 2.12. Statistical Analysis

Statistical analyses were conducted using SPSS version 22.0 (SPSS Inc., Chicago, IL, USA). Significant differences and interaction effects among sample groups were determined using two-way ANOVA, followed by Duncan’s multiple range test, with a significance level of *p* < 0.05. Pearson’s correlation coefficient was used for correlation analysis.

## 3. Results and Discussion

### 3.1. Fucoxanthin Content

Fucoxanthin is a carotenoid found in the chloroplasts of brown algae species, responsible for their characteristic brown to yellow hues. This compound accounts for more than 10% of the estimated total carotenoid production in nature. Several studies have highlighted its functional properties, including antitumor, antiobesity, antidiabetic, anti-inflammatory, and anticancer effects [22]. Fucoxanthin has been shown to have no side effects, making it a promising functional ingredient for use in food, cosmetics, and pharmaceutical products. In fact, various countries are already marketing food supplements containing seaweed extracts with standardized amounts of fucoxanthin [23].

**Figure 2:**
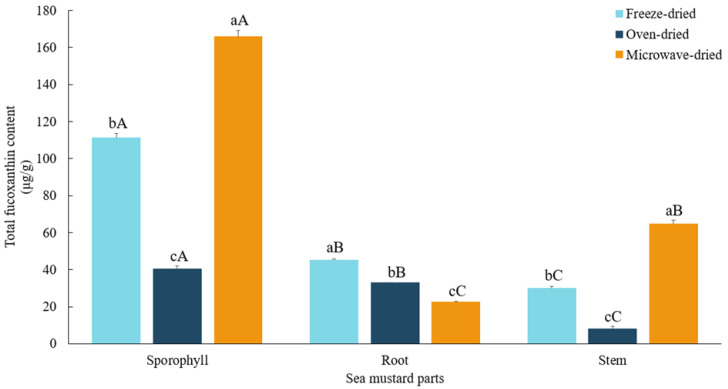
Total fucoxanthin content of sea mustard waste parts dried under different conditions. Small letters denote significant differences among different drying methods for each sea mustard part (a > b > c), while capital letters denote significant differences among the different parts of sea mustard for each drying condition (A > B > C) at the *p* < 0.05 level, according to Duncan’s multiple range test.

A significantly higher concentration of fucoxanthin (*p* < 0.05) was observed in microwave-dried sporophyll compared to other waste parts of sea mustard (Figure 2). Sporophyll consistently contains substantial amounts of fucoxanthin throughout the sea mustard harvest season [24]. Among the drying methods, microwave drying yielded the highest fucoxanthin content in both sporophyll (165.87 ± 3.22 μg/g) and stem (64.58 ± 2.1 μg/g), surpassing freeze-dried values by 49.2% and 114.9%, respectively. Similarly, in the study by Wibowo et al. [25], higher fucoxanthin content was reported in the brown seaweed *Padina australis* when subjected to high-temperature drying for shorter durations, compared to low-temperature drying with longer durations. Consistently, in this study, oven drying at a lower temperature (40 °C), which required a longer drying time compared to microwave drying, reduced fucoxanthin levels in both sporophyll and stem, retaining only 36.4% and 26.3% of the freeze-dried values, respectively. Fung et al. [24] reported a 51.8% reduction in fucoxanthin from *U. pinnatifida* after blanching for 1 min, followed by oven drying at 60 °C for 24 h. Prolonged drying times may increase exposure to air, light, and oxygen, accelerating fucoxanthin degradation.

In the case of root, the highest fucoxanthin content was observed in freeze-dried samples (45.08 ± 0.76 μg/g). Notably, oven drying was more effective than microwave drying in preserving fucoxanthin in root, resulting in only a 27.1% reduction compared to freeze-dried samples. In contrast, microwave drying caused the greatest loss of fucoxanthin in root, reducing its content by nearly half relative to freeze-dried samples. The differences observed in fucoxanthin retention across sea mustard parts, particularly in the root, can be attributed to the distinct morphological structures of each part. Furthermore, the formation of different fucoxanthin isomers in specific seaweed parts appears to depend on the reactions induced by the various drying methods [25].

### 3.2. Chlorophyll Contents

Chlorophyll is a green pigment found in seaweed that serves as the central reaction center, accessory pigment, and photoprotector in photosynthesis [26]. Numerous studies have demonstrated that chlorophyll and its derivatives exhibit antioxidant, antimutagenic, and cancer-preventive properties [27]. Its unique chemical structure, consisting of a porphyrin ring and a long hydrophobic side chain, enables it to scavenge free radicals and modulate cellular processes, potentially aiding in the prevention of various diseases [28]. In addition to its biological functions, chlorophyll is widely used as a natural colorant and additive in food to enhance sensory characteristics and as a health-promoting ingredient in pharmaceuticals. Marine algae typically contain four types of chlorophyll: Chl a, Chl b, Chl c, and Chl d.

**Figure 3:**
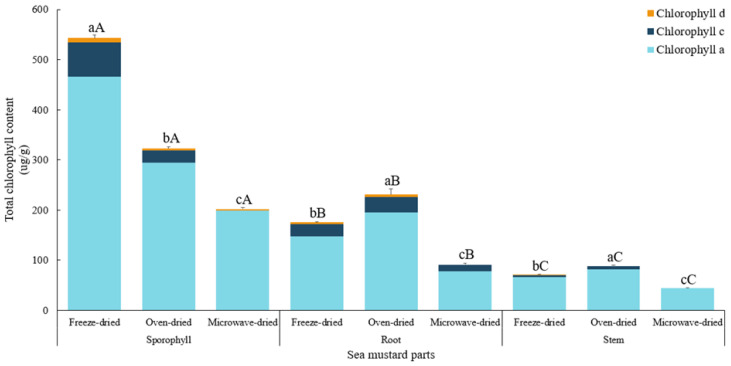
Chlorophyll contents of sea mustard waste parts dried under different conditions. Total vitamin chlorophyll content is equivalent to chlorophyll a + chlorophyll c + chlorophyll d. Small letters denote significant differences among different drying methods for each sea mustard part (a > b > c), while capital letters denote significant differences among the different parts of sea mustard for each drying condition (A > B > C) at the *p* < 0.05 level, according to Duncan’s multiple range test.

Among sea mustard waste parts, sporophyll exhibited the highest total chlorophyll content (*p* < 0.05) across all drying conditions: 543.2 ± 5.9 μg/g in freeze-dried samples, 322.61 ± 3.92 μg/g in oven-dried samples, and 196.74 ± 2.3 μg/g in microwave-dried samples (Figure 3). Sporophyll contained 1.4 to 3.1 times more chlorophyll than root and 3.7 to 7.6 times more than stem. The chlorophyll content of freeze-dried sporophyll was comparable to the value reported by Osorio et al. [13], where *U. pinnatifida* extracted with 90% acetone yielded 574.1 ± 33.2 μg/g of chlorophyll. The darker green color observed in sporophyll, both before and after the drying process, aligns with its higher chlorophyll content compared to root and stem (Figure 1). No Chl b and minimal Chl d were detected, as the primary chlorophylls in brown algae are Chl a and Chl c [29].

Regarding drying conditions, sporophyll recorded significantly higher chlorophyll levels (*p* < 0.05) when freeze-dried. Chlorophyll content decreased by 40.6% in oven-dried samples and by 63.8% in microwave-dried samples. Conversely, in root and stem, oven drying resulted in chlorophyll contents 31.3% and 23.5% higher, respectively, than freeze drying. Microwave drying, however, caused the most significant reduction in total chlorophyll content across all sea mustard parts, with sporophyll showing the greatest reduction (63.8%), followed by root (48.2%) and stem (38.6%) compared to freeze-dried values.

Both phenolic compounds and pigments, such as chlorophyll, are highly susceptible to oxidative degradation, especially at elevated temperatures. Chen and Roca [30] observed that heat treatment processes like boiling and microwaving reduced chlorophyll content in brown algae by approximately 30%, with Chl c being more severely affected than Chl a. Interestingly, unlike other edible seaweeds, chlorophyll a in brown seaweeds was not completely degraded under these conditions.

### 3.3. Total Antioxidant Activities

Sea mustard is widely recognized for its antioxidant properties [31]. While most studies have predominantly focused on the leaf, which is the most commonly consumed part, this research evaluated the antioxidant potential of its waste parts. The root, sporophyll, and stem exhibited significant levels of DPPH and ABTS radical scavenging activities. These radical scavenging agents neutralize peroxide radicals, thereby interrupting radical chain reactions and preventing oxidative damage.

The DPPH radical scavenging activity was significantly highest (*p* < 0.05) in freeze-dried roots (1582.37 ± 3.02 μg GAE/g), followed by sporophyll (922.18 ± 30.55 μg GAE/g) and stem (130.11 ± 4.33 μg GAE/g). Roots exhibited 1.4 to 2.8 times higher DPPH radical scavenging activity than sporophyll and 2 to 12.2 times higher than stem across all drying conditions. Similar trends were observed for ABTS radical scavenging activity, with freeze-dried roots showing the highest value (2.52 ± 0.03 mg AAE/g), followed by sporophyll (0.93 ± 0.02 mg AAE/g) and stem (0.54 ± 0.01 mg AAE/g). Root demonstrated ABTS activity 1.6 to 2.7 times higher compared to sporophyll, and 1.6 to 4.7 times higher compared to stem across all drying conditions. According to Wang et al. [32], *U. pinnatifida* contains various polyphenols and flavonoids, which exhibit potent antioxidant activities.

**Figure 4:**
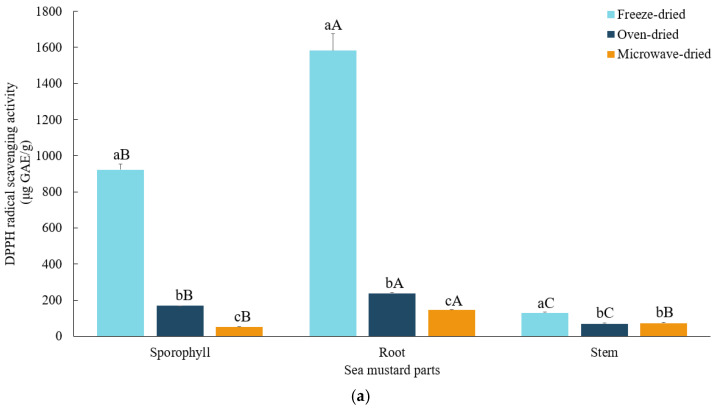

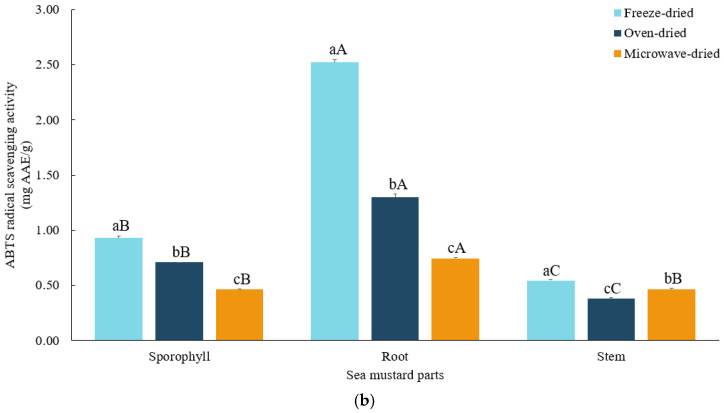
DPPH (**a**) and ABTS (**b**) radical scavenging activities of sea mustard waste parts dried under different conditions. The unit used for DPPH radical scavenging activity is micrograms of gallic acid equivalents per gram while the unit for ABTS radical scavenging activity is micrograms of ascorbic acid equivalents per gram. Small letters denote significant differences among different drying methods for each sea mustard part (a > b > c), while capital letters denote significant differences among the different parts of sea mustard for each drying condition (A > B > C) at the *p* < 0.05 level, according to Duncan’s multiple range test.

The different drying methods employed in this study significantly affected the antioxidant activities of sea mustard waste parts. DPPH and ABTS radical scavenging activities were both found to be significantly highest (*p* < 0.05) in freeze-dried samples (Figure 4a,b). Similar findings were reported by Badmus et al. [12], where freeze-dried samples of five species of brown seaweeds exhibited the highest antioxidant activities, while microwave-dried samples showed the lowest. Additionally, Subbiah et al. [33] observed consistently high phenolic content and antioxidant activities in freeze-dried Australian beach-cast brown seaweeds compared to oven-dried and vacuum-dried samples. Heat treatment notably reduced the DPPH radical scavenging activities. Root and sporophyll were the most affected, showing huge reductions from freeze-dried values of about 90.8% and 94% when microwave-dried, and 84.9% and 81.8% when oven-dried. For the stem, the reduction was more significant when oven-dried (46.9%) compared to microwave-dried (43.6%).

A similar trend was observed for ABTS radical scavenging activity, where both root and sporophyll showed approximately 70% and 50% reduction, respectively, when microwave-dried, with less reduction when oven-dried (48.5% and 23.7%, respectively). In contrast, the stem exhibited a lower retention rate when oven-dried compared to microwave-dried.

These results suggest that high temperatures can have adverse effects on the antioxidant activities of seaweeds. Oxidation of molecules with antioxidant activity may occur due to elevated temperatures [34]. In this study, the sea mustard waste parts were exposed to the highest temperatures during microwave drying. In contrast, thermo-sensitive antioxidants can be preserved in freeze drying due to its lower temperatures and limited exposure to oxygen.

### 3.4. Total Polyphenol Contents

Seaweed is rich in secondary metabolites, including phenolic compounds, which play a crucial role in reducing the risk of chronic diseases such as cancer, metabolic and neurodegenerative disorders, and cardiovascular diseases. These compounds mitigate the effects of reactive oxygen species (ROS) by functioning as reducing agents, hydrogen donors, singlet oxygen quenchers, and metal chelators [35]. Phenolic compounds in brown algae, such as catechins, flavonols, and phlorotannins, are widely used in health-promoting products. According to Holdt and Kraan [36], brown seaweeds contain higher concentrations of phenols than green and red seaweeds.

In this study, root exhibited the highest total polyphenol content (*p* < 0.05) among all samples, with 2718.81 ± 20.26 μg GAE/g in freeze-dried and 566.24 ± 12.69 μg GAE/g in microwave-dried conditions. Consistent with these findings, Park et al. [37] reported that subcritical water extraction of *U. pinnatifida* roots resulted in significantly higher polyphenol content and antioxidant activity compared to sporophyll and leaf extracts.

**Figure 5:**
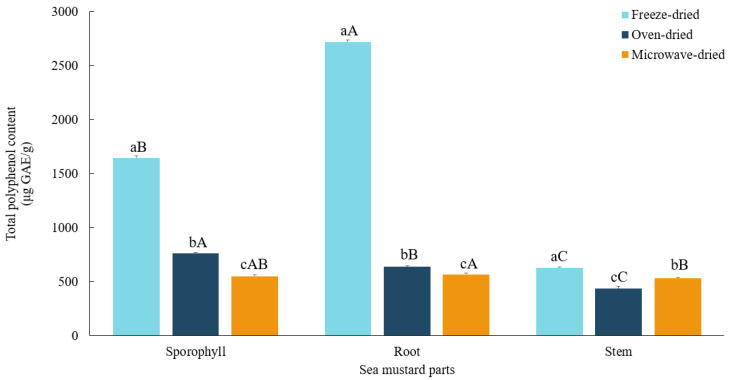
Total polyphenol contents of sea mustard waste parts dried under different conditions. The unit used is micrograms of gallic acid equivalents per gram. Small letters denote significant differences among different drying methods for each sea mustard part (a > b > c), while capital letters denote significant differences among the different parts of sea mustard for each drying condition (A > B > C) at the *p* < 0.05 level, according to Duncan’s multiple range test.

The drying method significantly influenced polyphenol retention. Freeze-dried sea mustard samples showed the highest total polyphenol content (*p* < 0.05) compared to oven-dried and microwave-dried samples (Figure 5). Elevated temperatures markedly reduced polyphenol content, with root and sporophyll losing 76.6% and 53.8% in oven drying and 79.2% and 66.6% in microwave drying, respectively. In contrast, stem exhibited a lower reduction in microwave drying (15.2%) compared to oven drying (31%).

Phenolic compounds are particularly susceptible to oxidative degradation under high temperatures, leading to significant losses during drying processes. Zhao et al. [38] reported that freeze-dried *Sargassum fusiforme* retained the highest polyphenol content, while hot air, microwave, and sun drying methods resulted in lower levels. Similarly, Badmus et al. [12] observed that freeze drying preserved higher polyphenol concentrations in *Fucus spiralis* and *Fucus serratus* compared to oven and microwave drying.

### 3.5. Total Flavonoid Contents

Flavonoids are phytochemicals abundant in plant foods, known for their potent antioxidant activities. Their chemical structure, featuring multiple hydroxyl (OH) groups on the benzene ring, confers significant radical scavenging capacity [39]. Beyond antioxidative properties, flavonoids exhibit diverse biological activities, including anti-inflammatory, antimutagenic, and anticarcinogenic effects, making them valuable for nutraceutical, pharmaceutical, medicinal, and cosmetic applications [40]. Seaweed extracts are recognized as natural flavonoid sources with proven health benefits and no reported side effects [41]. Identified flavonoids in *U. pinnatifida* leaf include rutin, caffeic acid, catechol, quercetin, and morin [42].

**Figure 6:**
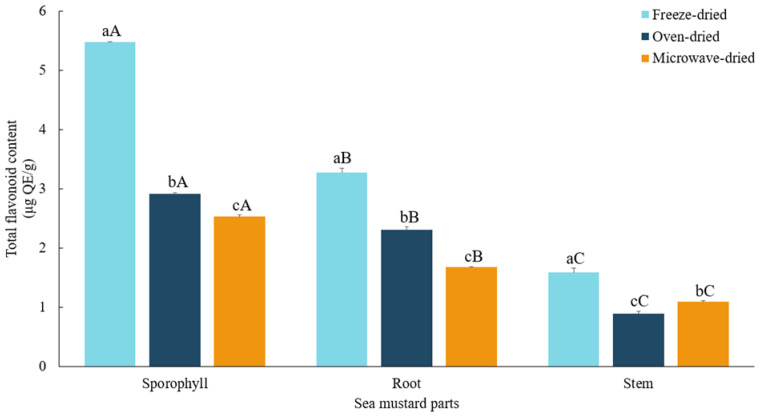
Total flavonoid contents of sea mustard waste parts dried under different conditions. The unit used is micrograms of quercetin equivalents per gram. Small letters denote significant differences among different drying methods for each sea mustard part (a > b > c), while capital letters denote significant differences among the different parts of sea mustard for each drying condition (A > B > C) at the *p* < 0.05 level, according to Duncan’s multiple range test.

In this study, significantly higher (*p* < 0.05) flavonoid content was found in freeze-dried sporophyll (5.47 ± 0.01 μg QE/g), followed by root (3.27 ± 0.07 μg QE/g) and stem (1.58 ± 0.08 μg QE/g) (Figure 6). Sporophyll had 1.3 to 1.7 times higher total flavonoid content compared to root and 2.3 to 3.5 times higher compared to stem across all drying methods. Despite its tough texture, sporophyll is rich in bioactive compounds with notable health benefits and is traditionally used in Chinese medicine [43].

Freeze-dried sea mustard samples exhibited the highest total flavonoid content (*p* < 0.05), surpassing oven-dried and microwave-dried samples. High-temperature drying methods significantly reduced flavonoid content. Oven drying retained more flavonoids in root and sporophyll, with reductions of 29.5% and 46.8%, respectively, compared to microwave-dried samples, which showed reductions of 48.8% and 53.8%. Conversely, stem exhibited a greater reduction in flavonoid content with oven drying (44.2%, nearly half of the freeze-dried value) compared to microwave drying (31.3%).

Flavonoids, a subclass of polyphenolic compounds, are particularly vulnerable to high temperatures. Subbiah et al. [33] demonstrated that freeze-dried *Ecklonia radiata* retained higher flavonoid content than oven-dried (40 °C) and vacuum-dried (70 °C) samples. Similarly, Uribe et al. [44] reported significant flavonoid degradation in *Durvillaea antarctica* at 40–50 °C. These findings highlight the critical role of low-temperature drying methods in preserving flavonoid content in seaweed.

### 3.6. Total Phlorotannin Contents

Phlorotannins, the primary antioxidants in brown seaweeds, play crucial roles in chemical defense, protection against oxidative damage, interactions with other organisms, and as integral components of the cell wall. These compounds naturally precipitate proteins and inhibit glucosidase and amylase, slowing the absorption of dietary carbohydrates [45]. According to Kumar et al. [46], phlorotannins exhibit diverse bioactivities, including antiallergic, antimicrobial, antioxidant, anticancerous, antidiabetic, and anti-inflammatory properties. Brown algae, such as *U. pinnatifida*, may contain approximately 15% phlorotannins in their dry matter [47].

**Figure 7:**
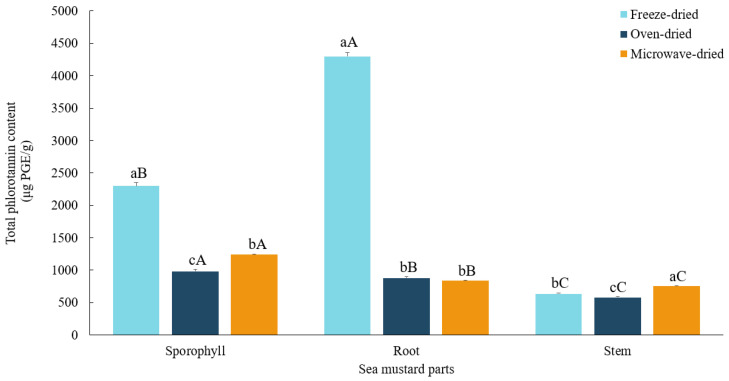
Total phlorotannin contents of sea mustard waste parts dried under different conditions. The unit used is micrograms of phloroglucinol equivalents per gram. Small letters denote significant differences among different drying methods for each sea mustard part (a > b > c), while capital letters denote significant differences among the different parts of sea mustard for each drying condition (A > B > C) at the *p* < 0.05 level, according to Duncan’s multiple range test.

In this study, root exhibited the highest phlorotannin content in freeze-dried samples (4298.22 ± 62.16 μg PGE/g), whereas sporophyll contained the highest levels in both oven-dried (985.22 ± 29.13 μg PGE/g) and microwave-dried (1243.14 ± 5.74 μg PGE/g) samples (Figure 7). Although sporophyll has been extensively studied for its phlorotannin content, limited data are available for root and stem. According to Dong et al. [46], sporophyll’s high phlorotannin content makes it a promising ingredient for functional foods and supplements. Moreover, phlorotannins from *U. pinnatifida* can enhance the gel properties of minced mackerel, improving texture [48].

Drying conditions significantly affected the total phlorotannin levels in sea mustard waste parts. Freeze drying preserved the highest total phlorotannin content, especially in roots and sporophyll. Root phlorotannin levels were notably reduced by 80% under both oven and microwave drying conditions. For sporophyll, microwave drying resulted in a lower reduction (45.9%) compared to oven drying (57.1%). In contrast, the stem exhibited an 18.3% higher phlorotannin content when microwave-dried compared to freeze-dried, while oven drying caused a slight 8.8% reduction relative to freeze drying.

The susceptibility of phlorotannins to degradation under high-temperature drying is consistent with previous findings. Chowdhury et al. [49] reported higher phlorotannin levels in freeze-dried *Ecklonia cava* compared to sun-dried or oven-dried samples, attributing the loss to heat and photo-oxidation processes. However, the effects of drying methods can vary across different seaweed parts due to differences in their physical and chemical composition. These variations can result in differential responses to heat exposure, resulting in distinct outcomes in terms of phlorotannin retention, as observed in the stem, where microwave drying resulted in the highest total phlorotannin levels.

### 3.7. Phytomenadione Contents

Phytomenadione, also known as vitamin K_1_ or phylloquinone, is a fat-soluble vitamin that functions as an essential cofactor for γ-glutamyl carboxylase, an enzyme responsible for the post-translational conversion of glutamate residues into γ-carboxyglutamate. This modification is crucial for the biological activity of γ-carboxylated proteins, which primarily play roles in blood coagulation and calcium homeostasis in bone tissue [50]. Vitamin K is primarily sourced from photosynthetic organisms, including a variety of vegetables and algae. Notably, edible seaweeds such as *S. fusiforme*, *U. pinnatifida*, and the red alga *Porphyra* sp. are abundant in phylloquinone [51].

**Figure 8:**
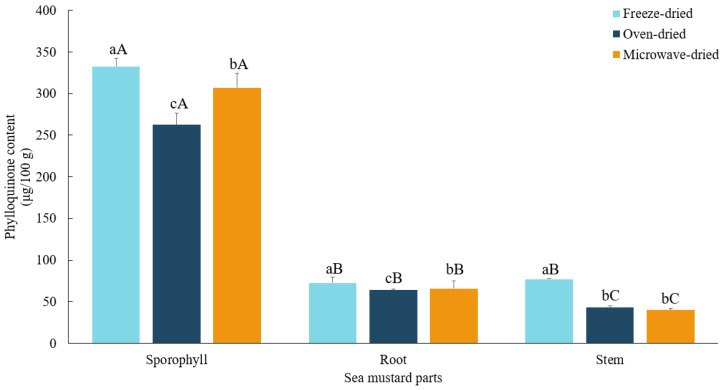
Phylloquinone contents (vitamin K_1_) of sea mustard waste parts dried under different conditions. The unit used is micrograms of phloroglucinol equivalents per gram. Small letters denote significant differences among different drying methods for each sea mustard part (a > b > c), while capital letters denote significant differences among the different parts of sea mustard for each drying condition (A > B > C) at the *p* < 0.05 level, according to Duncan’s multiple range test.

In this study, among the waste parts of sea mustard, the sporophyll exhibited the highest vitamin K content across all drying methods: 332.59 ± 9.82 μg/100 g in freeze-dried samples, 262.46 ± 14.12 μg/100 g in oven-dried samples, and 307.04 ± 17.71 μg/100 g in microwave-dried samples (Figure 8). The sporophyll contained 4.1 to 4.7 times more vitamin K than the root and 4.4 to 7.7 times more than the stem across all drying methods. These values exceed the vitamin K content of broccoli (102 μg/100 g), a well-established source of vitamin K_1_ [52]. The recommended daily intake of vitamin K is 120 μg for males and 90 μg for females [53].

Freeze-dried sea mustard waste parts consistently exhibited significantly higher (*p* < 0.05) vitamin K content compared to microwave-dried and oven-dried samples. In contrast to the other bioactive compounds analyzed, the reduction in vitamin K during microwave drying was minimal, with roots and stems losing only 8.9% and 7.7%, respectively, compared to freeze drying. Oven drying resulted in slightly higher losses, with reductions of 11.7% and 21.1% for roots and sporophyll, respectively. The stem, however, showed more significant reductions in vitamin K content, with losses of 44% and 47.9% for oven-dried and microwave-dried samples, respectively.

Vitamin K is relatively heat-stable, and high heat exposure can help release it. Damon et al. [54] demonstrated that boiling vegetables like broccoli and carrots led to higher vitamin K content. However, prolonged heat exposure may cause vitamin K degradation. In the study of Alibas et al. [55], microwave drying of basil leaves at 300 W resulted in higher vitamin K content compared to conventional drying at 50 °C. The retention rate of vitamin K was nearly 50% lower in the conventionally dried samples compared to those dried using a microwave.

### 3.8. Cobalamin Contents

Cobalamins, which exhibit vitamin B_12_ activity, have the most complex structure and the largest molecular weight among all vitamins [56]. While cobalamins are primarily synthesized by specific bacteria in the gastrointestinal tract of animals, making animal products such as meat, eggs, and milk the main dietary sources of vitamin B_12_, certain studies have suggested that seaweeds contain significant amounts of this vitamin. This is likely due to the presence of B_12_-synthesizing bacteria that colonize macrophytic algae in the photic zone from deeper waters [57].

In this study, a significantly higher amount of vitamin B_12_ (*p* < 0.05) was detected in sporophyll compared to stem across all drying methods. Both freeze-dried (332.59 ± 9.82 μg/100 g) and oven-dried (332.59 ± 9.82 μg/100 g) sporophyll samples showed elevated levels, while the stem exhibited lower concentrations (Table 2). This is consistent with the findings of Gil et al. [58], which showed that *U. pinnatifida* sporophyll contains higher concentrations of cobalamin than the leaf or blade. Notably, no detectable vitamin B_12_ was found in freeze-dried and oven-dried root, although microwave-dried root had the highest recorded level (332.59 ± 9.82 μg/100 g). This highlights that the drying method can significantly influence the cobalamin content in different parts of the seaweed in distinct ways.

The vitamin B_12_ content of sporophyll was not significantly altered by oven drying when compared with freeze-dried values. However, a substantial reduction (65.1%) in vitamin B_12_ was observed when microwave drying was applied. For stem, both drying methods resulted in significant reductions in vitamin B_12_ levels, with oven drying causing a decrease of approximately 44%, while microwave drying led to a more substantial loss of 75%.

Both sporophyll and stem exhibited notable amounts of vitamin B_12_ across all drying conditions, particularly in freeze-dried samples. These values surpass the vitamin B_12_ content found in whole milk (0.54 µg/100 g) and eggs (1.02 µg/100 g) as reported by the USDA Food Central [59,60] and are higher than the recommended daily intake of vitamin B_12_, which is 2.4 µg for adults [61].

Given that vegetarians and vegans are at a higher risk for vitamin B_12_ deficiency, as this vitamin is predominantly found in animal-derived foods, seaweed offers a viable alternative for meeting daily B_12_ requirements. Deficiency in vitamin B_12_ is associated with various health concerns, especially among non-meat eaters, pregnant women, and the elderly [62]. The high vitamin B_12_ content in seaweed, demonstrated in this study, suggests its potential as a valuable source of this essential nutrient. In line with this, Castillejo et al. [63] observed elevated vitamin B_12_ levels in smoothies supplemented with macroalgae, further supporting the role of seaweed in addressing vitamin B_12_ deficiency. Thus, sea mustard waste parts, rich in both vitamin B_12_ and other bioactive compounds, could serve as a promising source of nutritional and functional ingredients.

### 3.9. Correlation and Interaction Analyses

#### 3.9.1. Between Bioactive Compounds

**Figure 9:**
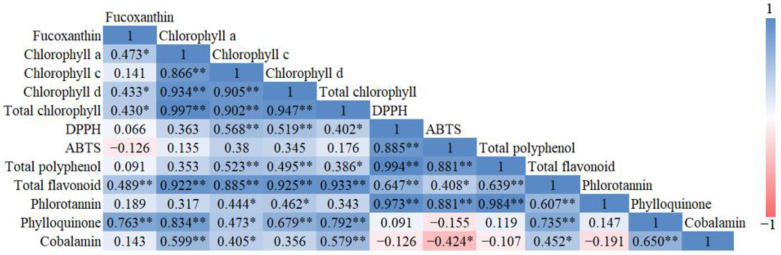
Correlation matrix between all the bioactive compounds analyzed in the study (*: *p* < 0.05, **: *p* < 0.01).

A positive correlation was observed between antioxidant activities and the total polyphenol, flavonoid, and phlorotannin contents of sea mustard waste parts (Figure 9). Polyphenols and flavonoids are natural antioxidants found in various forms and abundant quantities in many plants. Moreover, the pigments chlorophyll and fucoxanthin showed a positive correlation with total flavonoid content. Both pigments, like flavonoids, also exhibit antioxidant properties. Seaweeds are known to contain several bioactive compounds such as polysaccharides, polyphenols, vitamins and minerals which possess antioxidant properties. These antioxidant compounds inhibit oxidation reactions in the human body, which may potentially cause cell damage and lead to degenerative diseases, including cancer [23].

#### 3.9.2. Between Drying Methods and Sea Mustard Parts

The interactions between sea mustard parts and different drying conditions are presented in Table 3. All bioactive compounds analyzed in the study (including fucoxanthin, chlorophylls (a, b, and c), total chlorophyll content, DPPH and ABTS radical scavenging activities, total polyphenol, flavonoid, phlorotannin, phylloquinone, and cobalamin contents) showed highly significant interactions with drying conditions and sea mustard waste parts (*p* < 0.001). This indicates that the effect of the drying method is dependent on the specific part of the seaweed being analyzed.

**Figure 10:**
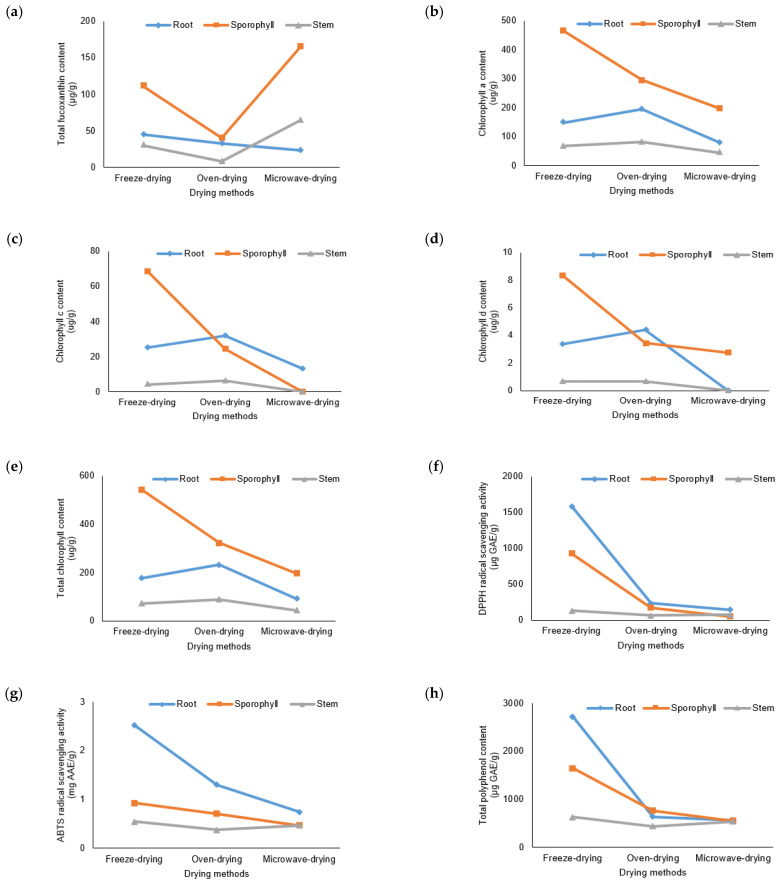

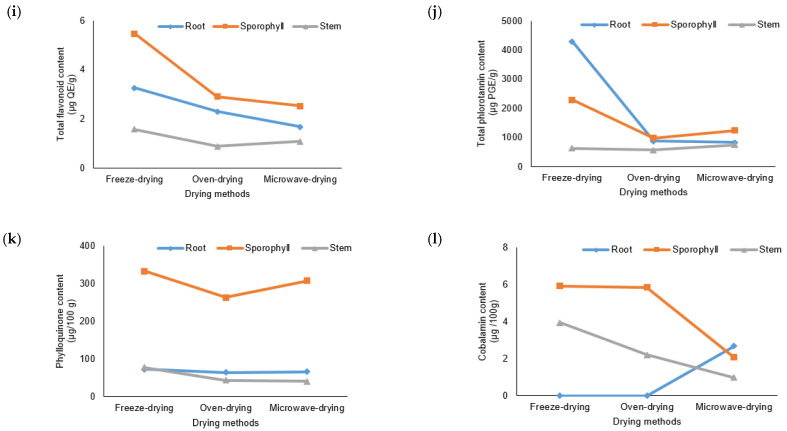
Interaction effects of drying method and sea mustard parts on various bioactive compounds and antioxidant activities: (**a**) total fucoxanthin content, (**b**) chlorophyll a, (**c**) chlorophyll c, (**d**) chlorophyll d, (**e**) total chlorophyll content, (**f**) DPPH radical scavenging activity, (**g**) ABTS radical scavenging activity, (**h**) total polyphenol content, (**i**) total flavonoid content, (**j**) total phlorotannin content, (**k**) phylloquinone content, and (**l**) cobalamin content.

As illustrated in Figure 10, the fucoxanthin, chlorophylls (a and c), and total chlorophyll contents of sporophyll exhibited greater variability in response to different drying methods compared to root and stem, which displayed more consistent trends. Similarly, sporophyll showed greater variation in total flavonoid and phylloquinone contents than root and stem. In contrast, root was more severely impacted by the drying methods in terms of DPPH and ABTS radical scavenging activities, as well as total polyphenol and phlorotannin contents, whereas sporophyll and stem displayed similar patterns across these parameters. For cobalamin content, the trends were distinct wherein sporophyll showed a significant reduction when microwave-dried, while root only exhibited detectable cobalamin levels in microwave-dried samples, demonstrating an opposing trend.

Overall, the results suggest that sporophyll and root were more greatly influenced by drying conditions, with the specific effects varying depending on the bioactive compound analyzed. These findings highlight the critical role of drying methods in preserving or altering the bioactive compound content in different seaweed parts.

## 4. Conclusions

The increasing global production of waste is a pressing issue, contributing significantly to the severity of global warming. Seaweed waste, which is typically discarded into the ocean, increases environmental pollution but has the potential to be transformed into functional ingredients for various industries. The present study evaluated the levels of bioactive compounds in different waste parts of *U. pinnatifida* (sporophyll, root, and stem) under three drying conditions: freeze drying, oven drying, and microwave drying.

The sporophyll, which exhibited the darkest green color among the samples, contained the highest concentrations of fucoxanthin and chlorophyll. It also had the highest levels of phytomenadione and cobalamin among the sea mustard waste parts. In contrast, the root exhibited the highest antioxidant activity, along with elevated levels of total flavonoids, polyphenols, and phlorotannins. Antioxidant activities were positively correlated with the total polyphenol, flavonoid, and phlorotannin contents, while chlorophyll and fucoxanthin levels were positively correlated with flavonoid content.

The bioactive compounds in sea mustard waste demonstrated strong antioxidant properties, with high levels of phytomenadione and cobalamin, particularly in the freeze-dried sporophyll and roots. However, drying methods significantly influenced the amount of bioactive compounds, depending on the specific part of the seaweed. Both the roots and sporophyll were more adversely affected by prolonged heat exposure compared to the stem, which can be attributed to the distinct structural matrices of each part.

Sustainable seaweed production requires efficient utilization of all parts, including waste. Dried seaweed waste can be processed into functional products, offering diverse applications based on consumer needs and industry demands.

## Figures and Tables

**Table 1:** The gradient condition of HPLC mobile phases for cyanocobalamin analysis.

Time (min)	Water (%)	Acetonitrile (%)
0	100	0
11	85	15
19	75	25
20	90	10
26	100	0
40	100	0

**Table 2:** Cobalamin contents (vitamin B_12_) of sea mustard waste parts dried under different conditions.

Drying Methods	Sea Mustard Parts	Vitamin B_12_ (µg/100 g)
Freeze drying (−70 °C)	Sporophyll	5.92 ± 0.53 ^aA(1)^
	Root	ND ^(2)^
	Stem	3.94 ± 0.12 ^aB^
Oven drying (40 °C)	Sporophyll	5.83 ± 0.28 ^aA^
	Root	ND
	Stem	2.21 ± 0.15 ^bB^
Microwave drying (260 W)	Sporophyll	2.07 ± 0.13 ^bB^
	Root	2.68 ± 0.24 ^aA^
	Stem	0.97 ± 0.08 ^cC^

^(1)^ Mean ± SD. Means with different small letters denote significant differences among different drying methods for each sea mustard part (a > b > c), while capital letters denote significant differences among the different parts of sea mustard for each drying condition (A > B > C) at the *p* < 0.05 level, according to Duncan’s multiple range test. ^(2)^ ND: not detected.

**Table 3:** F values for the effects of drying conditions and waste parts on bioactive compounds in sea mustard.

Parameters	Drying Conditions (A)	Sea Mustard Waste Parts (B)	A × B
1. Fucoxanthin	2.092	4.337	1236.419 ***
2. Chlorophyll a	2.093	9.412 *	1293.595 ***
3. Chlorophyll c	1.709	1.713	0.542 ***
4. Chlorophyll d	2.231	4.171	47.730 ***
5. Total chlorophyll content	2.036	7.065 *	1256.476 ***
6. DPPH radical scavenging activities	3.821	1.598	418.223 ***
7. ABTS radical scavenging activities	2.255	4.439	2134.889 ***
8. Total polyphenol contents	3.549	1.367	5400.135 ***
9. Total flavonoid contents	5.419	10.038 *	676.113 ***
10. Total phlorotannin contents	2.268	1.343	3230.805 ***
11. Phylloquinone content	3.415	184.719 ***	11.291 ***
12. Cobalamin content	0.358	2.609	225.853 ***

* *p* < 0.05, *** *p* < 0.001.

## Data Availability

The original contributions presented in the study are included in the article/Supplementary Materials, further inquiries can be directed to the corresponding author.

## Supplementary Materials

The following supporting information can be downloaded at: https://www.mdpi.com/article/10.3390/foods13233815/s1, Supplementary Figure S1: HPLC chromatograms of (a) vitamin K_1_ standard and (b) freeze-dried root sample; (c) vitamin B_12_ standard and (d) freeze-dried sporophyll sample.
