# Supplementary material for: Bioactive Compounds of Sea Mustard (Undaria pinnatifida) Waste Affected by Drying Methods
Source: Foods. 2024 Nov 26;13(23):3815. doi: 10.3390/foods13233815 (PMC11639787; doi:10.3390/foods13233815)
Supplement: Supplementary file 1 [file foods-13-03815-s001.zip › foods-3301144-supplementary.pdf]

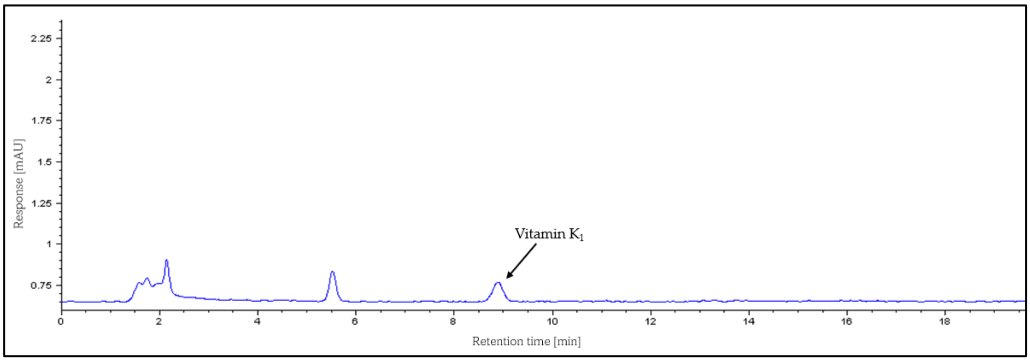

(**a**)

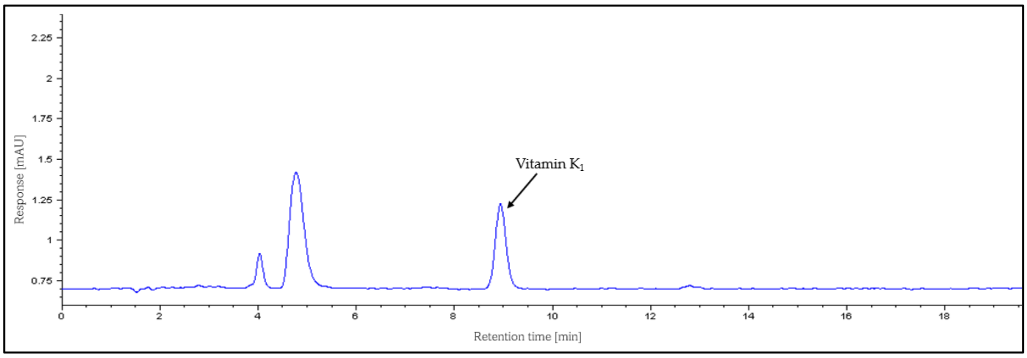

(**b**)

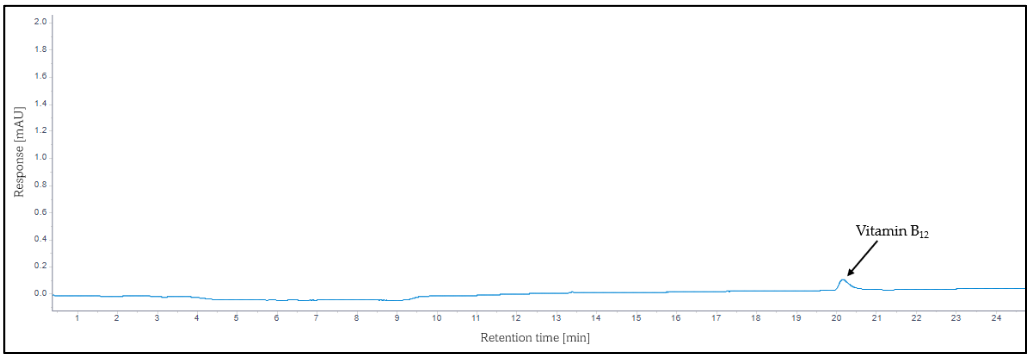

(**c**)

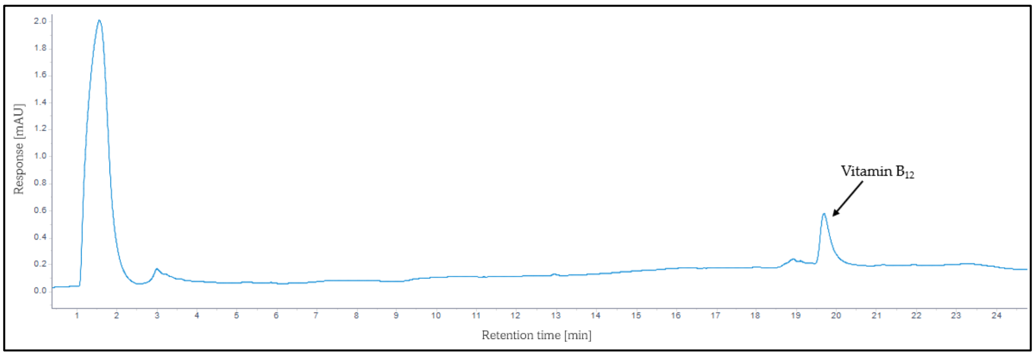

(**d**)

Supplementary Figure S1. HPLC chromatograms of (**a**) vitamin K_1 standard and (**b**) freeze-dried root sample; (**c**) vitamin B_{12} standard and (**d**) freeze-dried sporophyll sample.
